# Persistent immune abnormalities discriminate post-COVID syndrome from convalescence

**DOI:** 10.1007/s15010-023-02164-y

**Published:** 2024-02-07

**Authors:** Julia Sbierski-Kind, Stephan Schlickeiser, Svenja Feldmann, Veronica Ober, Eva Grüner, Claire Pleimelding, Leonard Gilberg, Isabel Brand, Nikolas Weigl, Mohamed I. M. Ahmed, Gerardo Ibarra, Michael Ruzicka, Christopher Benesch, Anna Pernpruner, Elisabeth Valdinoci, Michael Hoelscher, Kristina Adorjan, Hans Christian Stubbe, Michael Pritsch, Ulrich Seybold, Julia Roider, The Post COVID Care and KoCo19 study groups

**Affiliations:** 1 Department of Medicine IV, University Hospital, Ludwig-Maximilians-Universität München, Munich, Germany; 2 Department of Internal Medicine IV, Division of Diabetology, Endocrinology and Nephrology, University Hospital, Eberhard-Karls-Universität Tübingen, Tübingen, Germany; 3 The M3 Research Center, University Clinic Tübingen (UKT), Medical Faculty, Otfried-Müllerstr. 37, Tübingen, Germany; 4 Charité, Universitätsmedizin Berlin, Freie Universität Berlin and Humboldt- Universität Zu Berlin, Institute of Medical Immunology, Augustenburger Platz 1, 13353 Berlin, Germany; 5 Berlin Institute of Health (BIH) at Charité, Universitätsmedizin Berlin, BIH Center for Regenerative Therapies (BCRT), Charitéplatz 1, 10117 Berlin, Germany; 6 Department of Infectious Diseases, Department of Medicine IV, University Hospital, Ludwig-Maximilians-Universität München, Munich, Germany; 7 German Center for Infection Research (DZIF), Partner Site Munich, Munich, Germany; 8 Department of Medicine IV, Division of Clinical Pharmacology, University Hospital, Ludwig-Maximilians-Universität München, Munich, Germany; 9 Division of Infectious Diseases and Tropical Medicine, University Hospital, Ludwig-Maximilians-Universität München, Munich, Germany; 10 COVID-19 Registry of the LMU Munich (CORKUM), University Hospital, Ludwig-Maximilians-Universität München, Munich, Germany; 11 Department of Medicine III, LMU University Hospital, LMU Munich, Munich, Germany; 12 Department of Medicine II, University Hospital, Ludwig-Maximilians-Universität München, Munich, Germany; 13 Department of Psychiatry and Psychotherapy, University Hospital, Ludwig-Maximilians-Universität München, Munich, Germany

**Keywords:** Innate lymphoid cells, COVID-19, Post-COVID-19-syndrome, Immune activation, Tissue immunology

## Abstract

**Background:**

Innate lymphoid cells (ILCs) are key organizers of tissue immune responses and regulate tissue development, repair, and pathology. Persistent clinical sequelae beyond 12 weeks following acute COVID-19 disease, named post-COVID syndrome (PCS), are increasingly recognized in convalescent individuals. ILCs have been associated with the severity of COVID-19 symptoms but their role in the development of PCS remains poorly defined.

**Methods and results:**

Here, we used multiparametric immune phenotyping, finding expanded circulating ILC precursors (ILCPs) and concurrent decreased group 2 innate lymphoid cells (ILC2s) in PCS patients compared to well-matched convalescent control groups at > 3 months after infection or healthy controls. Patients with PCS showed elevated expression of chemokines and cytokines associated with trafficking of immune cells (CCL19/MIP-3b, FLT3-ligand), endothelial inflammation and repair (CXCL1, EGF, RANTES, IL-1RA, PDGF-AA).

**Conclusion:**

These results define immunological parameters associated with PCS and might help find biomarkers and disease-relevant therapeutic strategies.

**Supplementary Information:**

The online version contains supplementary material available at 10.1007/s15010-023-02164-y.

## Introduction

Viral infections can result in chronic symptoms that persist in previously healthy convalescent individuals across a wide range of viral families, including Ebola virus, influenza, Epstein–Barr virus, and dengue fever [1, 2]. The main symptoms are fatigue, exertion intolerance, sleep disturbances, neurocognitive and sensory impairment, flu-like symptoms, myalgia/arthralgia, and a plethora of nonspecific symptoms [3]. These post-acute infection syndromes (PAIS) are associated with autoimmunity and endothelial dysfunction, affecting both large and small vessels [3, 4]; however, risk factors and the underlying pathophysiology remain largely unknown.

The COVID-19 pandemic, caused by infection with severe acute respiratory syndrome coronavirus 2 (SARS-CoV-2), has led to an increasing prevalence of convalescent patients with prolonged and persistent sequelae following acute SARS-CoV-2 infection—known as ‘long COVID’ or ‘post-COVID syndrome’ (PCS) [5]. The estimated prevalence of PCS ranges from 5 to 50% [6], thus presenting an enormous global health burden, and can affect both patients with mild or severe forms of acute COVID-19 disease [7]. Clinical symptoms include fatigue, malaise, depression, cognitive impairment, persistent cough, dyspnea, palpitations, and headaches [8]. While the acute phase of COVID-19 has been extensively studied, providing health care professionals with efficient treatment options, the pathogenesis of PCS remains unclear, with current hypotheses including autoimmunity, latent virus reactivation, tissue, and endothelial damage [9].

The extreme respiratory distress in patients with acute COVID-19 is mediated primarily by immunopathology and systemic inflammation. Pathological immune signatures suggestive of T cell exhaustion, delayed bystander CD8^+^ T cell activation, and higher plasma Granulocyte–macrophage colony-stimulating factor (GM-CSF) and C–X–C motif chemokine ligand 10 (CXCL10) levels are associated with severity of the disease [10–12]*.* Survivors of severe COVID-19 show persistent immune abnormalities, including elevated levels of pro-inflammatory cytokines [13]. In addition to systemic inflammation, SARS-CoV-2 infects endothelial cells, causing virus-mediated apoptosis and consecutive endotheliitis and, thus, may promote endothelial damage and increased recruitment of activated immune cells into the endothelium and surrounding tissue [14].

Dysregulated respiratory CD8^+^ T cell responses may contribute to impaired tissue conditions and development of pulmonary sequelae [15]. Recent work identified persistent immunological dysfunction in patients with post-acute sequelae of COVID-19, including highly activated innate immune cells and marked differences in specific circulating myeloid and lymphocyte populations [16, 17]*.*

Innate lymphoid cells (ILCs) are tissue-resident effector immune cells with crucial roles in normal tissue development and remodeling [18, 19]. ILCs can be grouped into type 1, type 2, and type 3/17 *flavors* with associated cytokines (type 1—IFNγ; type 2—IL-4, IL-5, IL-9, IL-13; type 3/17—IL-17A/F, IL-22) that coordinate discrete spatial and temporal aspects of anti-microbial immune responses as well as organ development, homeostasis, and repair [20].

These cells also participate in both protective and pathologic immune responses during lung tissue perturbation [21, 22]*.* Several studies detected a reduction in total circulating ILCs in severe COVID-19 patients, while relative group 2 innate lymphoid cells (ILC2) levels, particularly NKGD^+^ ILC2s, were increased [23, 24]*.* Although ILCs appear central to lung infection and repair, their role in PCS remains critically unexplored.

Here, we used multicolor flow cytometry and multiplex cytokine assays on plasma from (1) healthy, uninfected controls (*n* = 32, ‘HC’); (2) previously SARS-CoV-2-infected probands in the convalescent phase without persisting symptoms (*n* = 32, convalescent controls, ‘CC’); and (3) patients with persisting symptoms following acute COVID-19 (*n* = 27, post-COVID, ‘PC’) to identify specific immunological alterations, including ILCs, in PCS. Most participants were non-hospitalized during acute SARS-CoV-2 infection and CC and PC individuals had persisting symptoms for more than 12 weeks from the initial infection. We found expanded circulating ILC precursors (ILCPs) in PC individuals while ILC2s were decreased. Patients with persisting symptoms also displayed elevated pro-inflammatory cytokines (interleukin (IL)-1RA, IL-1a), chemokines associated with trafficking of immune cells (Chemokine (C–C motif) ligand 19 (CCL19/MIP-3b), Fms-related tyrosine kinase 3 ligand (FLT3-Ligand)), and endothelial inflammation and repair (chemokine (C–X–C motif) ligand 1 (CXCL1), epidermal growth factor (EGF), Chemokine (C–C motif) ligand 5 (CCL5/RANTES), platelet-derived growth factor A (PDGF-AA)).

## Materials and methods

### Study design

#### post-covid-care study

The Post-COVID-Care (PCC) study is an ongoing prospective single-center study comprised of patients with persisting symptoms following acute COVID-19. Participants with COVID-19 sequelae were recruited from the post-COVID outpatient clinic at the Ludwig-Maximilian-University (LMU) University Hospital in Munich. Samples were collected from participants enrolled between April and July 2022. Peripheral blood mononuclear cells (PBMCs) isolated from blood samples were analyzed from 27 age- and sex-matched patients with persisting symptoms for more than 12 weeks following acute SARS-CoV-2 infection (PC group). Inclusion criteria were age ≥ 18 years; persisting symptoms > 12 weeks within 6 months following initial COVID-19 infection. None of the participants reported co-infections (e.g., bacterial superinfections) during acute SARS-CoV-2 infection. Pre-specified exclusion criteria were other explanations for the symptom onset or complete resolution of symptoms. All participants were scheduled for follow-up for at least 6 months and up to 24 months if symptoms persisted. At baseline and during the routine follow-up visits, blood samples were obtained and each patient completed progressive web app (PWA)-based questionnaires (LCARS-C, LMU Munich, https://github.com/hcstubbe/lcarsc). Patients who did not undergo any follow-up on site were asked to fill out the follow-up surveys using the PWA-based questionnaire at home using a computer, smartphone or tablet. Informed consent was obtained from all participants before inclusion into the study. Clinical characteristics of study participants are reported in Table 1. The study was approved by the Ethics Committee of the Medical Faculty at LMU Munich (No. 21-1165) and registered to the German Clinical Trials Register (DRKS-ID: DRKS00030974).

**Table 1:** Clinical and demographic characteristics of study cohorts

		Healthy controls (HC)	Convalescent controls (CC)	Post-COVID (PC)
*n*	Number	32	32	27
Sex				
Male	Number (%)	15 (46.9%)	14 (43.8%)	9 (33.3%)
Female	Number (%)	17 (53.1%)	18 (56.2%)	18 (66.7%)
Age (years)				
20–29	Number (%)	6 (18.8%)	6 (18.8%)	4 (14.8%)
30–39	Number (%)	10 (31.2%)	12 (37.5%)	10 (37.1%)
40–49	Number (%)	15 (46.9%)	12 (37.5%)	8 (29.6%)
> 49	Number (%)	1 (3.1%)	2 (6.2%)	5 (18.5%)
	Mean	36	36	37
BMI (kg/m^2^)				
< 18.5	Number (%)	3 (9.4%)	0 (0%)	0 (0%)
18.5–25	Number (%)	14 (43.8%)	22 (68.8%)	15 (65.2%)
25–30	Number (%)	10 (31.2%)	9 (28.1%)	5 (21.7%)
> 30	Number (%)	5 (15.6%)	1 (3.1%)	3 (13.1%)
	Mean	25.2	23.4	24*
Time from PCR to visit median (in days)			273 (min: 125; max: 318)	113 (min: 89; max: 292)
Disease severity				
Emergency hospitalization number (%)		n.a	1(3.1)	2 (7.4)
Severity score (0–5)	Median	n.a	3.0	n.a
Comorbidities				
No comorbidity	Number (%)	25 (78.1)	23 (71.5)	16 (59.3)
Coronary heart disease	Number (%)	1 (3.1)	0 (0)	2 (7.4)
Diabetes mellitus	Number (%)	0 (0)	0 (0)	1 (3.7)
Obesity	Number (%)	1 (3.1)	1 (3.1)	3 (11.1)
COPD/asthma	Number (%)	5 (15.6)	6 (18.8)	3 (11.1)
(Ex) smoker	Number (%)	6 (18.8)	12 (32.5)	5 (18.5)

Data are given as numbers (percentages). *BMI* body mass index. Sex, age and BMI were comparable between groups with an overall mean age of 36 years, 58% females and BMI of 24.2 kg/m^2^

There were no significant differences in the proportion of male or female participants between groups (*p* = 0.5530 [Chi-square: 1.185, *d*.*f*. = 2]). Participants were well matched in age (Kruskal–Wallis post hoc *p* = 0.9276) and BMI (Kruskal–Wallis post hoc *p* = 0.3315)

*BMI was unknown for 4 individuals from the PC group

#### KoCo19-Shield study for control samples

For this project, two different control groups were used: (1) “CC-group”: seropositive SARS-CoV-2-convalescent patients without persisting symptoms (*n* = 32) and (2) “HC group”: seronegative individuals without any previous contact to SARS-CoV-2 (*n* = 32). Samples for these controls were derived from previously established cohorts and selection was performed to achieve optimal age and sex match with the PC group.

The KoCo19-Shield study cohort was originally established within a previously described population-based SARS-CoV-2 cohort study (KoCo19) [25, 26] to study SARS-CoV-2-specific immune responses in convalescent individuals > 3 months post-infection. Individuals from households with at least one person who had a PCR confirmed SARS-CoV-2 infection were contacted by the responsible official authorities (City of Munich Health department) in May and June 2020 and were recruited as previously described [27]. Individuals who expressed interest in participating were enrolled between September 29, 2020, and January 27, 2021. Furthermore, randomly selected 40 households from the KoCo19 study were selected as controls. In total, 36 households comprising 85 eligible members agreed to participate and were recruited during January 6–27, 2021. Participants of the control group did not show any seropositive tests for SARS-CoV-2 at baseline or during follow-up. PBMCs isolated from blood samples were analyzed from 32 age- and sex-matched seropositive convalescent patients without persisting symptoms (CC group) and from 32 controls without previous contact to SARS-CoV-2 (HC group). Personal data of the study participants were collected as previously described [25]. Participants with SARS-CoV-2 infection were asked to report date of symptom onset and acute disease severity, SARS-CoV-2 polymerase chain reaction (PCR) diagnostic testing results, and antibody testing results. All participants were also asked to provide SARS-CoV-2 vaccination status. Clinical demographics of study participants are reported in Table 1. The study was approved by the Ethics Committee of the Medical Faculty at LMU Munich (20–275 V) and the protocol is available online (www.koco19.de) [27]. Informed consent was obtained from all enrolled participants. The study is registered to the German Clinical Trials Register (DRKS-ID: DRKS00022155).

#### Blood sample processing

Peripheral blood samples from all participants were collected in four potassium-EDTA-coated blood collection tubes (Sarstedt) and were immediately processed at University Hospital, LMU, Munich, Germany. Whole blood was centrifuged at 450 × g for 10 min at room temperature (RT). Plasma was then transferred to 1.8-ml polyethylene Cryotube™ vials (ThermoFisher), aliquoted, and stored at − 80 °C. For isolation of PBMCs, two tubes each of the remaining whole blood sample were pooled and filled up to a total volume of 32.5 ml with Hank's Balanced Salts Solution (Capricorn or Sigma). 13.5 ml Histopaque®-1077 (Sigma) was added at the bottom of each tube and samples were centrifuged at 450 × g for 30 min at RT without break. PBMC layer on top of the Histopaque® layer was collected and washed twice in Hank’s balanced salts solution. Isolated cells were counted using a CASY cell counter and analyzer (Schärfe System GmbH) before storage in liquid nitrogen at − 180 °C for cryopreservation.

#### Flow cytometry

Cryopreserved PBMCs were thawed in a 37 °C water bath, pipetted into Iscove's Modified Dulbecco's Medium (IMDM) supplemented with 10% FCS medium, and washed by centrifugation. Three to six million cells per sample were incubated with antibodies to surface antigens (Table S1) for 30 min at 4 °C, washed with FACS buffer (1XDPBS, 3% FCS, 0.05% NaN3), fixed with 2% paraformaldehyde for 10 min, washed again with FACS buffer, and resuspended in FACS buffer. Samples were acquired on a BD LSRFortessa X-20. Fluorochrome compensation was performed with single-stained UltraComp eBeads (Invitrogen, Cat# 01–2222-42). To exclude debris, FSC-A/SSC-A gating was used, followed by FSC-H/FSC-A gating to select single cells and Zombie NIR fixable to exclude dead cells. Innate lymphoid cells were identified as lineage negative (CD1a^−^, CD14^−^, CD19^−^, CD34^−^, CD94^−^, CD123^−^, FcER1a^−^, TCRab^−^, TCRgd^−^, BDCA2^−^), CD45^+^, CD161^+^, CD127^+^, as indicated. The full gating strategy is shown in Fig. S1 and was adapted from previous work [28]. Data were analyzed using FlowJo version 10.7 software (TreeStar, USA) and compiled using Prism (GraphPad Software). T-distributed stochastic neighbor embedding (t-SNE) visualization of flow cytometry data was performed using Cytobank.

#### Quantification of plasma cytokine levels

Forty-six plasma cytokines (G-CSF, PDGF-AA, EGF, PDGF-AB/BB, VEGF, GM-CSF, FGF, GRZB, IL-1A, IL-1RA, IL-2, IL-27, IL-4, IL-6, IL-10, IL-13, TNF, IL-17C, IL-11, IL-18, IL-23, IL-6RA, IL-19, IFN-B, IL-3, IL-5, IL-7, IL-12p70, IL-15, IL-33, TGF-B, IFN-G, IL-1B, IL-17, IL-17E, CCL3, CCL11, CCL20, CXCCL1, CXCL2, CCL5, CCL2, CCL4, CCL19, CXCL1, CXCL10, PD-L1, FLT3, TACI, FAS, LEPTIN R, APRIL, OPN, BAFF, LEPTIN, BMP4, CD40 LIGAND, FAS LIGAND, BMP7, BMP2, and TRAIL) were analyzed using a Luminex platform (Human Cytokine Discovery, R&D System, Minneapolis, MN) according to the manufacturer’s instruction.

#### Unsupervised data analysis

Cytobank [29] was used for initial manual gating of Lineage-negative cells and ILC subsets group 1 innate lymphoid cell (ILC1), ILC2, and ILCP, using the same gating strategy as described above. Lineage-negative cells were subjected to dimensionality reduction using Cytobank opt-SNE with default hyperparameters and following embedding markers with normalized scales Cytobank arcsinh transformation: CD117, CD127, CD161, CD45RA, CD56, CRTH2, HLA-DR, and SLAMF1. All pre-gated events were used without prior downsampling from 91 samples. To perform downstream statistical analyses in R (http://www.r-project.org/) and visualize t-SNE maps across the 91 samples, events within ILC subsets were exported from Cytobank as tab-separated values containing compensated and transformed marker expression levels as well as t-SNE coordinates and metacluster assignment. T-SNE plots were generated after subsampling each sample to contain a maximum of 2500 events. High-resolution group differences were visualized by calculating Cohen's D for a given comparison across the t-SNE map. We used the probability binning algorithm available through the R *flowFP* package [30] and generated adaptive 2D histograms. A single binning model was created on collapsed data from all samples, by recursively splitting the events at the median values along the two t-SNE dimensions. We chose a grid of 256 bins to have on average, at least eight cells per bin in each sample for statistical accuracy. Since there was a significant difference between cellular frequency distributions between the six measurement days, the batch effect was first regressed out by fitting a linear model to each bin after applying the arcsine-square-root transformation for proportions. The group-difference effect sizes were then calculated for each bin using the cohen.d function of the *effsize* package. To get a smoothed representation of the effect size map, adaptive binning was performed on a series of rotated coordinates and per cell-averaged effect size values were used to color-encode each cell throughout the t-SNE map. All analyses were performed using R version 4.1.1, available free online at https://www.r-project.org.

### Statistical analysis

The sample size was not pre-determined through formal power analysis. Data were analyzed using Prism version 8 (GraphPad Software, La Jolla, CA). All column graphs are presented as means ± standard error of the mean (SEM) unless otherwise noted with * = *p* < 0.05, ** = *p* < 0.01, *** = *p* < 0.001, **** = *p* < 0.0001. For comparisons between three groups, one-way ANOVA with Tukey´s multiple comparisons test was used. For comparison of age and BMI between study groups, one-way analysis of variance with Kruskal–Wallis and Dunn’s correction for multiple comparisons were performed (see Table 1). For comparison of age between study groups, a Chi-squared test was used (see Table 1). Correlation analyses were performed using Pearson’s correlation coefficient. Each symbol reflects individuals for flow analysis or plasma cytokine levels.

## Results

### Clinical characteristics of study participants

**Fig. 1:**
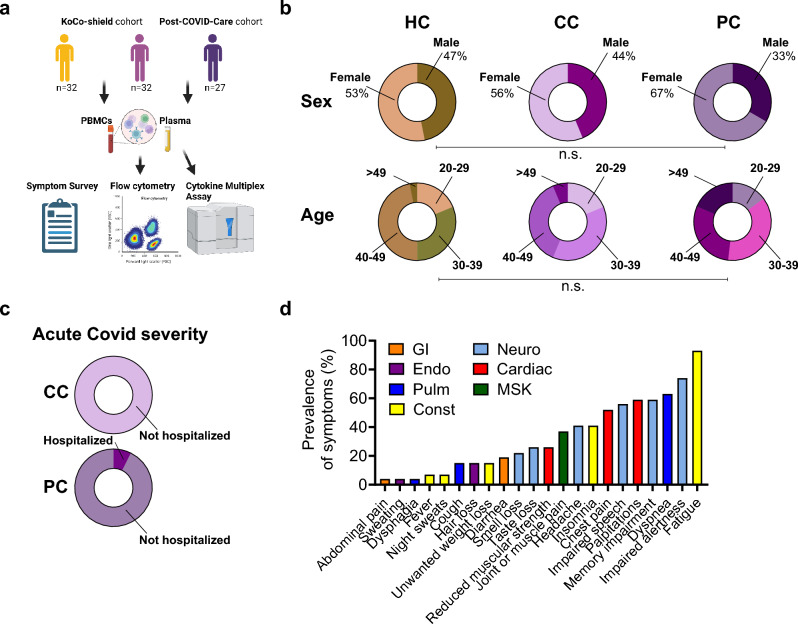
Clinical characteristics of study cohorts. **a** Overview of study cohorts and methods. The figure is partly created with BioRender.com. **b** Demographic data for healthy, uninfected controls (HC), convalescent SARS-CoV-2 participants without persisting symptoms (CC) and convalescent SARS-CoV-2 participants with persisting symptoms (PC) displayed as ring charts. Statistical significance is shown by capped lines as Chi-square tests for ‘Sex’ and post hoc comparisons for ‘Age’. Further characteristics are detailed in Table 1. **c** Percentage of hospitalization during acute COVID infection for CC and PC participants displayed as ring charts. **d** Prevalence of top 22 self-reported symptoms in PC participants (least prevalent (left) to most prevalent (right)). Symptoms are colored according to physiological systems. Gastrointestinal (GI), endocrine (Endo), pulmonary (Pulm), constitutional (Const), neurological (Neuro), cardiac, and musculoskeletal (MSK)

Patients, enrolled in the Post-COVID-Care study at the LMU University Hospital Munich, presented with persisting symptoms for more than 12 weeks following acute SARS-CoV-2 infection (PC group; *n* = 27) and were compared to convalescent patients without persisting symptoms (CC group; *n* = 32) and ‘healthy controls’ without previous contact to SARS-CoV-2 (HC group; *n* = 32), enrolled in the KoCo19-Shield sub study (Fig. 1a). Clinical demographics of both study cohorts are reported in Table 1.

The PCS, convalescent, and ‘healthy control’ groups were well matched in sex (67% female PC; 56% female CC; 53% female HC; *p* = 0.5530 [Chi-square: 1.185, *d*.*f*. = 2]), age (mean 37.15 years old PC; mean 36.09 years old CC; mean 35.91 years old HC; Kruskal–Wallis post hoc *p* = 0.9276), and BMI (mean BMI PC group 24.0 kg/m^2^; mean BMI CC group 23.4 kg/m^2^; mean BMI HC group 25.2 kg/m^2^; Kruskal–Wallis post hoc *p* = 0.3315) (Fig. 1b and Table 1). Only two patients with COVID-19 sequelae were hospitalized during acute infection, whereas none of the convalescent study participants were hospitalized (Fig. 1c), reflecting that some patients experience long-term health-consequences after acute COVID-19, regardless of disease severity. Consistent with numerous previous reports of PCS, the most common reported symptoms included constitutional symptoms, such as fatigue (93%) and insomnia (41%), and neurological symptoms, such as impaired alertness (74%), memory impairment (59%), and impaired speech (56%). Cardiac symptoms, including palpitations (59%), chest pain (52%), and reduced muscular strength (26%) were also a common complaint (Fig. 1d).

### Circulating ILCPs are elevated in PC patients with concurrent decrease in ILC2s

**Fig. 2:**
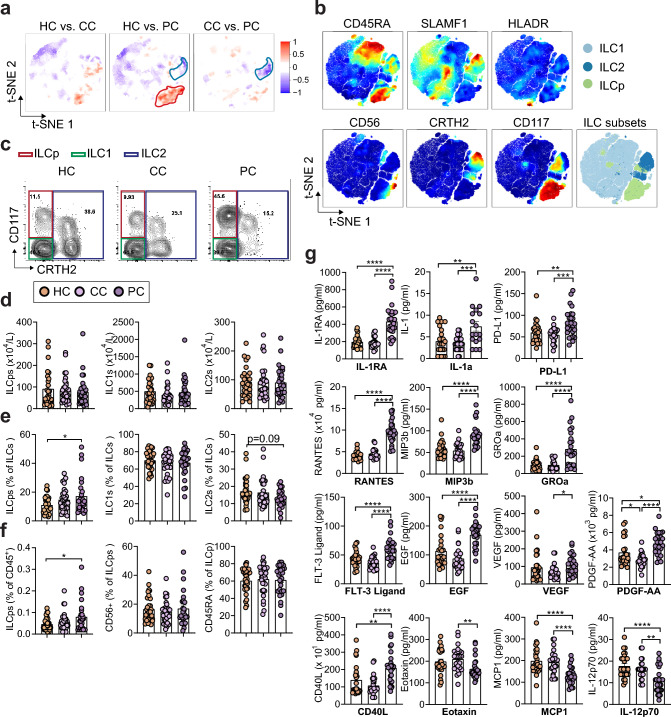
Post-COVID participants show altered cytokine expression and levels of innate lymphoid cells. **a** High-dimensionality reduction analysis of innate lymphoid cells (ILCs, gated as lymphocytes, singlets, and CD45^+^CD3^−^Lin^−^CD127^+^ cells as shown in Suppl. Fig. 1) from peripheral blood mononuclear cells (PBMCs) of HC, CC, and PC groups. High-resolution group differences were visualized by calculating Cohen's D for a given comparison across the t-SNE map. Residual plot showing differences between maps. Phenotypes within red circles were confirmed to be statistically more common in PC samples, and phenotypes within blue circles were less common in PC samples. Analysis is based on flow cytometry data from 32 HC, 32, CC, and 27 PC samples. **b** Relative expression intensities (combined HC, CC, and PC samples) of parameters used in the t-SNE analysis. **c**–**f** Representative flow cytometry plots (**c**) and quantification (**d**–**f**), showing total numbers (**d**) and percent (**e**, **f**) innate lymphoid cell populations in HC, CC, and PC groups at 3–10 months after acute COVID infection. **g** Multiplex assay quantification showing plasma levels of IL-1RA, IL-1a, PDL-1, RANTES, MIP-3b, Groa, FLT3 Ligand, EGF, VEGF, PDGF-AA, CD40L, Eotaxin, MCP1, and IL-12p70 in healthy controls with no prior SARS-CoV-2 infection (HC), convalescent SARS-CoV-2 participants without persisting symptoms (CC), and convalescent SARS-CoV-2 participants with persisting symptoms (PC) at 3–10 months after acute COVID infection

To investigate circulating ILC levels via flow cytometry in PCS, convalescent and ‘healthy controls’, we used a well-established gating strategy [28] (Suppl. Figure 1). Lin^−^CD127^+^ ILC subsets were defined as CD117^−^CRTH2^−^ ILC1s, CD117^+^ ILC progenitors (ILCP) [31], and CRTH2^+^ ILC2s. We used CD56 as a marker of activated or ILC3/NK cell-committed ILCP and CD45RA for naïve ILCP [28]. Recent work discovered CD45RA^+^ naïve-like ILCs, lacking proliferative activity, indicative of cellular quiescence [32]. To visualize multiple dimensions in simple two-dimensional plots and compare flow cytometry data between groups, we used stochastic neighbor embedding analysis (Fig. 2a, b). We found increased expression of the ILCP marker CD117 in PC compared to HC groups, while CRTH2 (marker for ILC2s) was decreased in PC compared to both CC and HC groups (Fig. 2a, b). However, the expression of proteins associated with ILC activation, CD56 (also defining NK cells with intermediate or high expression levels) and HLA-DR, was not different between groups (Fig. 2a, b). Next, we evaluated total numbers and frequencies of circulating ILCs and NK cells in patients with persisting symptoms after COVID-19 infection as compared to convalescent patients and healthy controls. We did not observe significant changes in total ILCs and subsequent ILC subsets (ILC2s, ILC1s, ILCPs) in PC compared to CC and HC groups (Fig. 2c, d, Suppl. Figure 2a). However, PC patients had significantly expanded levels of ILCPs with concurrent decreased ILC2 frequencies, while ILC1 levels remained unchanged (Fig. 2e). The role of Bar graphs indicate mean (± SE), *n* = 27–32 individuals per group, one-way ANOVA with Tukey’s multiple comparisons test (D, E, F), *p ≤ 0.05, **p ≤ 0.01, ***p ≤ 0.001, ****p ≤ 0.0001. See also Suppl. Fig. 2 and Suppl. Fig. 3.

ILC2s in viral-induced lung pathogenesis remains controversial. Although increased levels of IL-18, IL-13, and IL-6 have been reported along with accumulation of ILC2s during acute COVID-19, increased circulating ILC2s in moderate but not severe COVID-19 patients were found in other studies [33], consistent with their attrition by interferon (IFN)-γ in type 1 (viral-induced) inflammation [21]. Thus, ILC2s might have important roles in tissue repair during viral-induced epithelial cell damage, perhaps through crosstalk with other ILC subsets.

Recent work suggested that human ILCPs can interact with endothelial cells, fostering the adhesion of other innate and adaptive immune cells by stimulating pro-inflammatory cytokine expression of adhesion molecules. This activation occurs through the tumor necrosis factor receptor- and RANK-dependent engagement of Nuclear factor kappa-light-chain-enhancer of activated B cells (*NF-κB)* pathway [34]*.* ILCP levels as percentage of all CD45^+^ leukocytes were also increased in PC patients compared to the HC group (Fig. 2f). Nevertheless, PC patients did not show significant changes in CD45RA^+^ ILCPs, although CD56^+^ ILCPs were trending upwards, suggesting a circulating ILCP expansion without overt altered activation (Fig. 2f). Surprisingly, the expression of CD45RA was increased in ILC1 subsets in the PC group compared to HC and CC groups, while CD45RA^+^ ILC2 subsets remained unchanged (Suppl. Figure 2b), suggesting the increase of a quiescent local reservoir for the generation of differentiated ILCs [32]. Frequencies of HLA-DR^+^ ILC1s, percentages of CD117^+^ ILC2s, and the transcriptional expression of Signaling lymphocytic activation molecule 1 (SLAMF1) within the ILC2 compartment were similar between PC, CC, and HC groups (Suppl. Figure 2b). We could not find significant differences in NK cell frequencies between patients with PCS, convalescent, and healthy controls (Suppl. Figure 2c). We also did not find significant differences between frequencies of CD4^+^ or CD8^+^ T cells or regulatory T cells (data not shown). Together, these data indicate that ILCPs expand in patients with COVID-19 sequelae, without alteration of their activation state.

### Pro-inflammatory cytokines and growth factors are elevated in PCS

In COVID-19 patients with severe disease, cytokine storm and uncontrolled inflammatory responses, including endothelial inflammation and associated tissue damage, are recognized as one of the driving immunopathological features that can lead to death [10]. To uncover the immunological dysregulation in PCS, we quantified 46 molecular analytes in the plasma of patients from the CC and PC groups > 3 months after acute SARS-CoV-2 infection using a multiplex cytokine assay and compared them to healthy controls. Four key pro-inflammatory cytokines (IL-8, IL-6, interleukin-1 receptor antagonist (IL-1Ra) and IL-1a) were elevated in the PC group compared to the CC group; IL-1RA and IL-1a levels were also significantly higher in the PC group compared to healthy controls (Fig. 2g, Suppl. Figure 3a), while no difference was observed in transforming growth factor alpha (TGF-α), IL-7, IL-5, IL-4, IL-13, tumor necrosis factor (TNFα), IFN-γ and IL-1β (Suppl. Figure 3b). IL-10 was also elevated in the PC group compared to the CC group (Suppl. Figure 3c). IL-8 has been previously associated with a prothrombotic neutrophil phenotype in severe COVID-19 and blocking IL-8 signaling reduced SARS-CoV-2 spike protein-induced, human Angiotensin-converting enzyme 2 (ACE2)-dependent pulmonary microthrombosis in mice [35]. Surprisingly, levels of IL-8 were lower in CC compared to HCs, whereas other pro-inflammatory cytokines were not different between these groups (Suppl. Figure 3a, b). IL-1Ra was 2.16-fold higher in the PC group compared to the HC group and 2.22-fold higher compared to the CC group; other pro-inflammatory cytokines were only slightly increased (Fig. 2g, Suppl. Figure 3b). Importantly, programmed death-ligand 1 (PD-L1) was increased in the persisting symptom group compared to both convalescent and healthy control groups, consistent with previous reports, highlighting the prognostic role of sPD-L1 in COVID-19 patients [36] (Fig. 2g). Several chemokines (RANTES, MIP-3b, CXCL1) and growth factors (FLT3 Ligand, EGF, vascular endothelial growth factor (VEGF), PDGF-AA), that could be associated with trafficking of immune cells (MIP-3b, FLT3-Ligand) and endothelial inflammation (CXCL1, EGF, RANTES, PDGF-AA), and CD40L were also elevated in PC participants compared to both CC and HC groups (Fig. 2g). Interestingly, Eotaxin (CCL11), monocyte chemoattractant protein 1 (MCP1), and IL-12p70 were decreased in PC patients compared to both convalescent and healthy controls (Fig. 2g); some of these chemokines were associated with severe cases of acute COVID-19 [37]. The frequencies of plasma TNFα, FLT3-Ligand and CXCL1 (Groa) were positively correlated with levels of ILCPs (Suppl. Figure 4a–c), whereas PDGF-AA was negatively correlated with levels of naïve CD45RA^+^ ILCPs (Suppl. Figure 4d), indicating a strong coregulation of pro-inflammatory markers with activated ILCPs. Together, these data suggest persisting immune abnormalities in patients suffering from post-acute sequelae of COVID-19.

## Discussion

Persistent sequelae following acute COVID-19 are increasingly recognized in convalescent individuals. Our exploratory analyses identified immunological differences in patients with PCS as compared to well-matched convalescent and HC individuals at > 3 months post-infection. We found significant changes in circulating ILC subsets, including increased ILCPs and concurrent decreased ILC2 levels. In addition, pro-inflammatory cytokines (IL-1RA, IL-1a), chemokines associated with trafficking of immune cells (CCL19/MIP-3b, FLT3-Ligand) and endothelial inflammation and -repair (CXCL1, EGF, RANTES, PDGF-AA) were elevated in PC participants. We also observed an association between frequencies of circulating ILCPs and plasma markers associated with (endothelial)—inflammation and tissue repair. A limitation of our study is that for PC and CC groups, elapsed days since initial SARS-CoV-2 infection were different from acute disease (113 days for PC group vs. 273 days for CC group, data not shown); however, initial enrollment and collection of blood for immunophenotyping took place more than 3 months after onset of COVID-19 and none of the convalescent participants reported persisting symptoms after acute disease. Several studies have shown that pro-inflammatory cytokines remained significantly elevated in PC patients at month 8 after acute infection [17]. Acute SARS-CoV-2 infections within the PC group occurred in the period when the Omicron BA.2 variants were dominant (between January and March 2022), whereas participants of the convalescent group were confirmed to be infected with SARS-CoV-2 between March and April 2020, when parental strains drove the majority of new cases. While several risk factors, including comorbidities and virus variants, have been identified for the development of PCS [38], clinical symptoms are similar for different SARS-CoV-2 strains, with the exception of musculoskeletal pain, where chronic burden may be lower for Omicron compared to Delta variants [39]. Our work does not dissect how ILCPs or other activated innate and adaptive immune cells, contribute mechanistically to endothelial dysfunction in PCS. However, ILCP expansion along with elevated markers for endothelial inflammation in PC supports their interaction with endothelial cells; thereby facilitating enhanced inflammatory responses and endotheliitis in several organs. These findings may not only be interesting for long-term sequelae of COVID-19, but also for other viral infections that can result in PAIS in convalescent individuals. Further exploration of immunological alterations in PCS may delineate mechanisms of ILC-endothelial cell crosstalk and lead to disease-relevant targeted therapies.

### Supplementary Information

Below is the link to the electronic supplementary material.

Supplementary file1 (DOCX 1374 KB)

Supplementary file2 (PDF 90 KB)

## Data Availability

Further information and requests for resources and reagents should be delivered to and will be fulfilled by the Lead Contact, Julia Sbierski-Kind (Julia.Sbierski-Kind@med.uni-tuebingen.de).
