# Supplementary material for: Persistent immune abnormalities discriminate post-COVID syndrome from convalescence
Source: Infection. 2024 Feb 7;52(3):1087–97. doi: 10.1007/s15010-023-02164-y (PMC11142964; doi:10.1007/s15010-023-02164-y)
Supplement: Supplementary file 1 — Supplementary file1 (DOCX 1374 KB) [file 15010_2023_2164_MOESM1_ESM.docx]

**Supplementary Material**


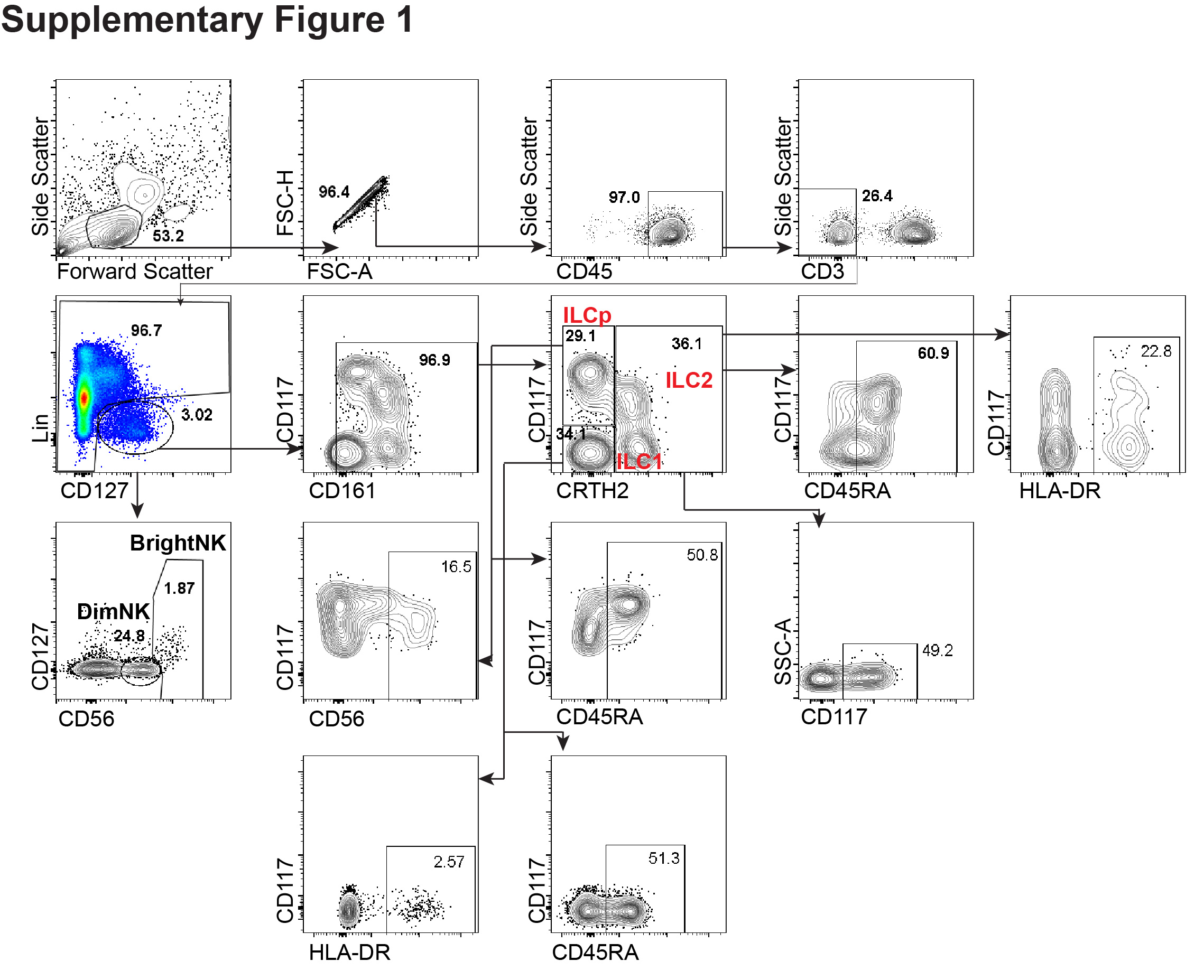


**Supplementary Figure 1: Flow cytometry gating scheme.**

Representative flow cytometry gating scheme for analysis of innate lymphoid cells (ILCs) in peripheral blood mononuclear cells (PBMCs). Numbers represent percentages of cells in respective gates. Lineage markers contained CD1a, CD14, CD19, CD123, BDCA2, FceR1, CD34, CD94, TCRαβ, TCRγδ, FceR1, and dead cell marker.

**
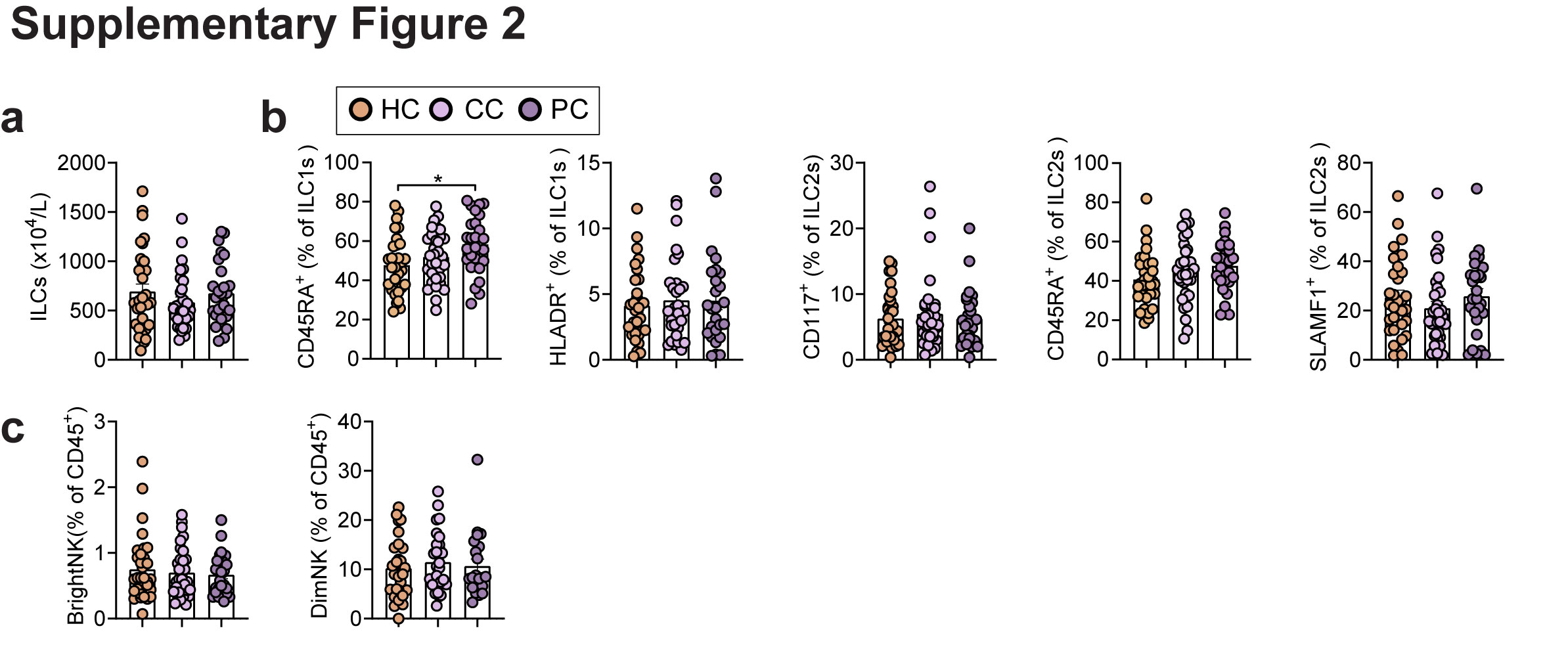
**

**Supplementary Figure 2: Levels of innate lymphoid cells among study groups.**

(a-c) Flow cytometry quantification, showing total numbers (a) and percent (b, c) of innate lymphoid cells (ILCs) and NK cells in peripheral blood mononuclear cells (PBMCs) of healthy controls with no prior SARS-CoV-2 infection (HC), convalescent SARS-CoV-2 participants without persisting symptoms (CC) and convalescent SARS-CoV-2 participants with persisting symptoms (PC) at 3-10 months after acute COVID infection.

Bar graphs indicate mean (±SE), one-way ANOVA with Tukey´s multiple comparisons test (A-C), *p ≤ 0.05.

**
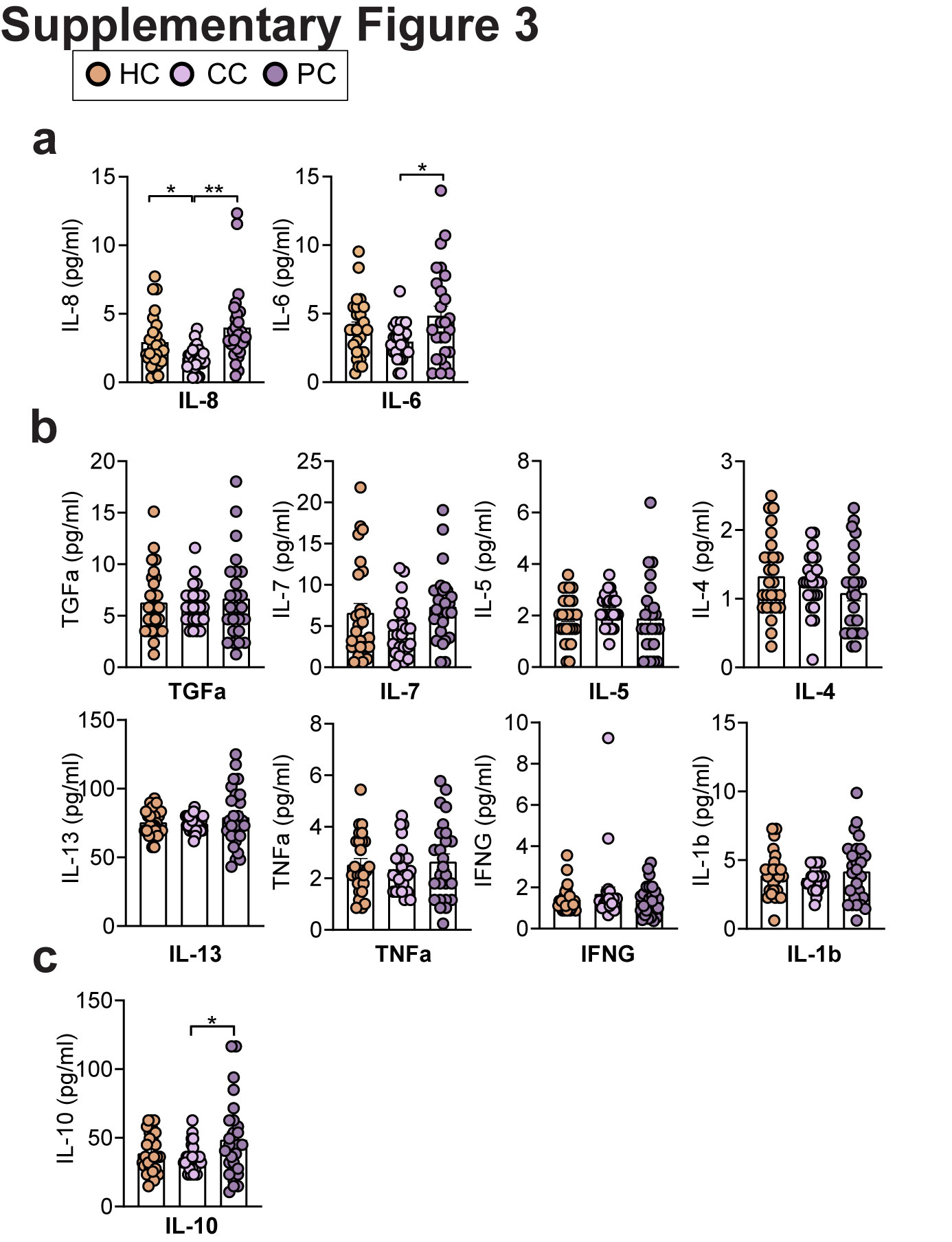
**

**Supplementary Figure 3: Post-COVID participants display altered plasma cytokine expression levels.**

(a-c) Multiplex assay quantification showing levels of IL-8, IL-6 (a), TGFa, IL-7, IL-5, IL-4, IL-13, TNFa, IFNG, IL-1b (b), and IL-10 (c) in plasma of healthy controls with no prior SARS-CoV-2 infection (HC), n=32, convalescent SARS-CoV-2 participants without persisting symptoms (CC), n=32, and convalescent SARS-CoV-2 participants with persisting symptoms (PC), n=27 at 3-10 months after acute COVID infection.

Bar graphs indicate mean (±SE), one-way ANOVA with Tukey’s multiple comparisons test, *p ≤ 0.05, **p ≤ 0.01.


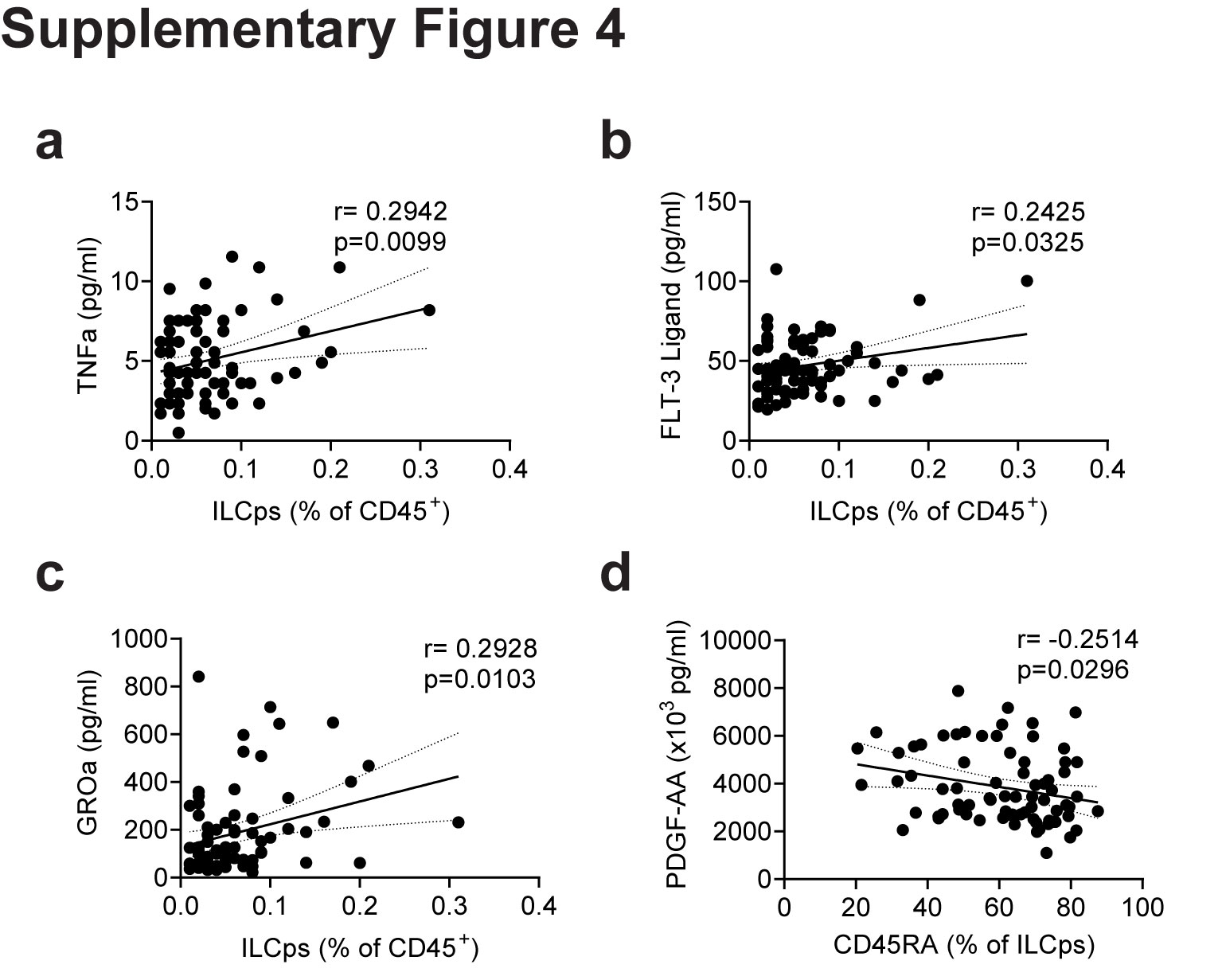


**Supplementary Figure 4: The percentage of circulating ILCps is associated with plasma cytokines involved in immune cell trafficking and proliferation.**

(a-d) Correlation of percentages of ILCps of leukocytes (a-c) or percentages of CD45RA^+^ ILCps (d) with levels of TNFa (a), FLT3-Ligand (b), CXCL1 (Groa) (c), and PDGF-AA (d) in plasma of healthy controls with no prior SARS-CoV-2 infection (HC), n=32, convalescent SARS-CoV-2 participants without persisting symptoms (CC), n=32, and convalescent SARS-CoV-2 participants with persisting symptoms (PC), n=27 at 3-10 months after acute COVID infection. The *r* indicates the Pearson correlation coefficient.
